# Polypyrrole-Modified Nanocellulose Exhibits Superior Performance for Hg(II) Adsorption

**DOI:** 10.3390/polym15122735

**Published:** 2023-06-19

**Authors:** Qizhong Xiong, Lei Zhang, Zijun Zhu, Gang Xu, Jianyuan Jing, Weifeng Zhang, Chaochun Zhang, Xinxin Ye

**Affiliations:** 1 Anhui Province Key Lab of Farmland Ecological Conservation and Pollution Prevention, Anhui Province Engineering and Technology Research Center of Intelligent Manufacture and Efficient Utilization of Green Phosphorus Fertilizer, College of Resources and Environment, Anhui Agricultural University, Hefei 230036, China; 2 Key Laboratory of JiangHuai Arable Land Resources Protection and Eco-Restoration, Ministry of Natural Resources, College of Resources and Environment, Anhui Agricultural University, Hefei 230036, China

**Keywords:** CNC, CNC@PPy, Hg(II) adsorption, agricultural waste

## Abstract

Cellulose, a kind of polymer containing abundant functional groups, has widespread use in the adsorptive removal of environmental pollutants. An efficient and environmental friendly polypyrrole (PPy) coating approach is employed to modify the agricultural by-product straw derived cellulose nanocrystal (CNC) into excellent property adsorbents for removing the heavy metal ion of Hg(II). The FT-IR and SEM-EDS results demonstrated that PPy is formed on the surface of CNC. Consequently, the adsorption measurements proved that the obtained PPy-modified CNC (CNC@PPy) possesses a remarkably enhanced Hg(II) adsorption capacity of 1095 mg g^−1^, owing to a plentiful functional group of doped Cl element on the surface of CNC@PPy by forming Hg_2_Cl_2_ precipitate. The results of the study suggest that the Freundlich model is more effective than the Langmuir model at describing the isotherms, while the pseudo-second order kinetic model is better suited to correlating with the experimental data compared to the pseudo-first order model. Further, the CNC@PPy exhibits an outstanding reusability, capable of maintaining 82.3% of its original Hg(II) adsorption capacity after five successive adsorption cycles. The findings of this work reveal a method to convert the agricultural by-product into high performance environmental remediation materials.

## 1. Introduction

Cellulose, as the plentiful reproducible natural polysaccharide, is the most valuable crude resource obtained from agricultural by-products for use by human beings [1,2]. Cellulose has been widely used in paper, materials, food, medicine, energy, and environmental renovation fields owing to its advantages of low cost, reproducibility, easy functionalization, and non-toxicity [3,4,5]. The fundamental skeleton of cellulose contains *β*-D-glucose with −CH_2_OH functional groups successively above and below the molecular planar of cellulose [6]. The abundant functional groups (e.g., hydroxyl) combined with the high porosity and relatively high specific surface area enable the cellulose to be an underlying adsorbent for contaminants governing fields, such as small organic molecule pollutants and heavy metal ions [7,8]. However, the extensive intra- and intermolecular hydrogen bonds in the undecorated natural cellulose slow the adsorption activity and selectivity of cellulose towards heavy metal ions by impeding the absorptivity of hydroxyl and ether groups [4,9].

Water contamination with heavy metal ions has gradually become the most engaging environmental and health problem in recent decades [10,11,12,13,14]. There are many strategies which have been used for removing pollutants in wastewater, including ion exchange, photo/electrochemical reduction, coagulation, chemical precipitation, adsorption, or membrane separation [15,16]. Among these approaches, the adsorption method, as a promising technology for environmental remediation, has attracted widespread attention with a series advantages, such as being eco-friendly, reusable, low cost, and easy to operate [17,18,19]. Thus, the adsorbents play a significant role during the adsorption process. In recent years, in order to improve the adsorption properties of un-artificial cellulose for heavy metal ions removal, vast research efforts have been made to introduce adsorption groups with high selectivity to functionalize the original cellulose through a series modification methods such as acidification, etherification, grafting polymerization, cross-linking, and esterification [2,16,20,21,22,23]. Among these reported functionalization strategies, PPy has been widely investigated for enhancing its adsorption capacity towards heavy metal ions owing to its merits of inexpensiveness, ease of polymerization, and environment durability [24]. A survey of investigations into the removal of heavy metal ions exhibits the impact of PPy as an highly effective material for capturing poisonous ions (e.g., Hg(II), Pb(II)) from polluted water [25,26]. Du and co-workers prepared PPy/MLS nano composites with a maximum adsorption capacity of 343.64 mg g^–1^ at 25 °C for removing Cr(VI) [27]. Chen prepared the PPy modified corn using the FeCl_3_ oxidation approach, and the obtained adsorbent of PPy/corn exhibited a saturated adsorption capacity of 84.7 mg g^–1^ for Cr(VI) [21]. Maity et al. reported a PPy composite containing a thiol-functionalized chelating group for the removal of Hg(II), with a maximum adsorption capacity of 1736.8 mg g^–1^ at 25 °C [28]. Despite the enhanced adsorption property of PPy-related materials for some heavy metal ions (e.g., Cr(VI), Hg(II)) having been proved, the adsorption capabilities are limited as yet and therefore it is still important to develop other approaches for boosting the removal of heavy metals.

In this study, we design and experimentally confirm a facile functionalization method via a standard chemical polymerization of pyrrole on the surface of a cellulose nanocrystal under acidic conditions to prepare the PPy-modified composite (CNC@PPy). The as-prepared CNC@PPy adsorbent possesses a high ability and selectivity for removing of Hg(II), with a resulting adsorption capacity of 1095 mg g^–1^ and a stability of up to 82.3% of the original adsorption capacity. The study findings indicate that the Freundlich model provides a better fit to the isotherms than the Langmuir model, and the pseudo-second order kinetic model is more closely aligned with the experimental data than the pseudo-first order model. We hope that the findings presented in this work pave the way for the development of cellulose-based high-performance adsorbents from agricultural by-products.

## 2. Materials and Methods

### 2.1. Chemicals

Sulfuric acid (H_2_SO_4_), mercuric nitrate (Hg(NO_3_)_2_), cadmium nitrate (Cd(NO_3_)_2_), lead nitrate (Pb(NO_3_)_2_), cupric nitrate (Cu(NO_3_)_2_), sodium chlorite (NaClO_2_), hydrogen peroxide (H_2_O_2_), potassium hydroxide (KOH), and hydrochloric acid (HCl) were purchased from Sinopharm Chemical Reagent Co., Ltd., Shanghai, China. Pyrrole monomer was provided by the Aladdin Industrial Corporation. All chemicals were used without further purification in the experiments.

### 2.2. Fabrication of CSC 

The corn straw cellulose (CSC) was extracted from the corn straw according to a previously reported approach [29,30]. The detailed extraction procedure was as follows: 1000 mg corn straw was added to a mixture solution with the presence of 80 mL benzene and 160 mL ethanol to dewax via a refluxed approach for 7.0 h. Then, the pre-prepared glacial acetic acid acidified sodium chlorite solution was used to dissolved lignin in CSC (pH of 3.0) at 75 °C for 1 h, which was repeated at least four times to obtain a white product. The above product was purified to remove hemicelluloses, residual starch, and pectin with 0.35 M KOH at 30 °C for about 24 h. Subsequently, the obtained product (100 mg) was successively purified by an acetic acid acidified sodium chlorite solution (200 mL, pH of 3.0), and 1.0 M KOH (200 mL) at 80 °C for 2 h, respectively. Lastly, the above extractive (100 mg) was further treated with 0.3 M HCl (250 mL) at 80 °C (for 2 h) and washed with deionized water to acquire highly purified CSC. The final CSC product was freeze-dried and stored for further use.

### 2.3. Fabrication of CNC and CNC@PPy

The CNC was synthesized from CSC with an improvement method [30,31]. The 10 g corn straw cellulose was hydrolyzed in 180 mL sulfuric acid (6.5 M) at 45 °C for 1 h with vigorous stirring. The cellulose suspension was then diluted with cold de-ionized (DI) water (ca. 10 times the volume of the acid solution used) to stop the hydrolysis, and allowed to settle overnight. The clear top layer was decanted and the remaining cloudy layer was centrifuged. The supernatant was decanted and the resulting thick white suspension was washed 3 times with DI water to remove all the soluble cellulose materials. The thick white suspension obtained after the last centrifugation step was placed inside dialysis membrane tubes (12,000–14,000 molecular weight cut-off) and dialyzed against slow running DI water for 7 days. The membrane tubes containing the extracted cellulose materials were placed periodically in DI H_2_O, and the procedure was continued until the pH of the water became constant over a period of one hour. The suspension from the membrane tubes was dispersed using ultrasound treatment in a Fisher Sonic Dismembrator (Fisher Scientific) for 5 min at 80% power. The final dispersed suspension product was freeze-dried to obtain cellulose nanocrystals and then diluted to the desired concentration for further uses.

The CNC@PPy was prepared by the polymerization of the pyrrole monomer on the surface of CNC via a hydrothermal approach in a mixture solution containing 20.0 μL of 36.5 wt% HCl, 1.0 mL of pyrrole monomer (14.0 mmol), and 3.0 mL 30 wt% H_2_O_2_ at 150 ℃ for 4 h. The CNC@PPy was obtained from the final black solution by using a centrifugation technique. 

### 2.4. Characterization

The morphology images were reported using field emission scanning electron microscopy (FESEM, SU8020). Elemental mapping analysis was performed in an energy-dispersive X-ray spectroscope (EDS) attached to SEM. The chemical component of as-prepared samples was characterized by X-ray photoelectron spectroscopy (XPS, ESCALAB 250) equipped with Al Kα_1,2_ monochromatized radiation at a 1486.6 eV X-ray source. Nitrogen adsorption–desorption isotherms were carried out with a Micromeritics ASAP 2020 physisorption analyzer. The Fourier transform infrared (FT-IR) spectra were carried on a Nicolet-Nexus FT-IR spectrometer. The concentration of Hg(II) was performed with an Inductively Coupled Plasma Optical Emission Spectroscopy (ICP-OES) from Thermo Fisher, Waltham, MA, USA, (Thermo iCAP 6300).

### 2.5. Adsorption Measurement

The Hg(II) stock solution (1000 mg L^–1^) was prepared by dissolving an appropriate amount of Hg(NO_3_)_2_ in deionized water. The concentrations of Hg(II) were determined by ICP-OES. For the isothermal adsorption experiments, the conical flasks with the absorbents and Hg(II) solution were placed in a shaker (300 rpm) for a fixed time, followed by filtration to remove the adsorbent. Samples were withdrawn when the adsorption reached equilibrium, and centrifuged. The amount of adsorbed Hg(II) per unit mass of the adsorbent was calculated as follows:

Q = (C_0_ – C_e_)V/M (1)

where Q (mg g^–1^) is the equilibrium adsorption capacity; C_0_ and C_e_ (mg L^–1^) are the initial and equilibrium concentrations of Hg(II) in solution, respectively; V (L) is the volume of the solution; and M (g) is the mass of adsorbents. The initial solution’s impact on adsorption was assessed under an optimized pH value of 5.0, and the temperature’s effect was examined at 25 ℃. The solution’s pH was adjusted by employing 0.1 M HCl solutions.

The CNC@PPy recycle experiments use the same procedure as described above. Between each consecutive recycle, the CNC@PPy adsorbents were regenerated by immersing in a 1.0 M HCl solution (100 mL) and shaking at 300 rpm by using a shaking thermostatic bath at 50 °C for 1 h.

All adsorption tests were performed in triplicate and the average values are reported. Firstly, CNC@PPy (20 mg) was immersed in 100 mL of each metal ion solution (initial concentration = 1 × 10^−3^ mol L^−1^, pH = 5), and the solution was stirred for 3 h at room temperature. Then, the adsorbent was filtered out. The concentrations of each metal ion after adsorption were measured with ICP-OES. 

Competitive adsorption tests were also performed. In these tests, the initial concentrations of each metal ion (Hg^2+^, Cd^2+^, Pb^2+^, Cu^2+^,) were fixed at about 1.5 × 10^−3^ mol L^−1^. Again, 20 mg CNC@PPy was immersed into 100 mL of a mixed-ion solution (pH = 5) and stirred for 3 h. The concentrations of each metal ion before and after adsorption were determined by ICP-OES.

Absorption kinetics: CNC@PPy (20 mg) was soaked in 50 mL of Hg^2+^ solution (200 mg g^−1^, pH = 5). After stirring for the desired time at room temperature, the adsorbent was filtered out and the metal ion concentration in the remaining solution was measured with ICP-OES.

Absorption isotherm: CNC@PPy(20 mg) was equilibrated with each 50 mL Hg^2+^ solution at different initial concentrations (10–200 mg L^−1^). The above mixture was stirred for 3 h to ensure that the adsorption had reached equilibrium. Then, the concentration of Hg^2+^ in each solution was measured using ICP-OES.

The details of the isotherm equations and kinetic equations are exhibited in the Supplementary Materials. 

## 3. Results and Discussion

**Figure 1:**
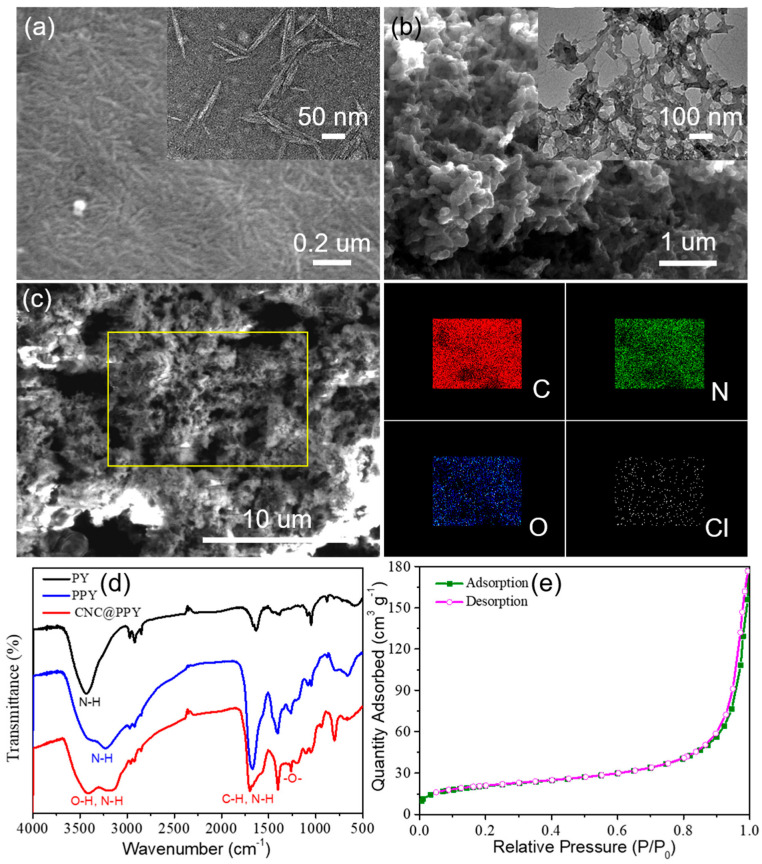
(**a**) SEM images of CNC. (**b**) TEM images of CNC@PPy. (**c**) SEM-EDS elemental mappings of CNC@PPy. (**d**) FT-IR spectra of Py, PPy, and CNC@PPy. (**e**) N_2_ adsorption–desorption isotherms of CNC@PPy.

The traditional hydrothermal reaction was employed to synthesize the core–shell structured CNC@PPys adsorbent with the presence of H_2_O_2_ to facilitate this reaction, as preliminarily exhibited in the schematic of Figure 1a. The FESEM and TEM images of obtained CNCs are exhibited in Figure 1a–c. As is shown, the collected CNC possesses an obvious nanofiber structure, which was randomly packed. In contrast to CNC, CNC@PPy revealed an irregular and rough state after coating with PPy, which should be advantageous for grabbing heavy metal ions (Figure 1a–c). To prove the successful coating of PPy on the surface of CNC, the SEM-EDS-based elemental mapping of CNC@PPy was observed (Figure 1a–c), which displayed a uniform distribution of C, N, O, and Cl elements without any other elements detected. The Cl element was introduced by applying the HCl solution as an acid catalysis reagent, and then obtaining Cl-doped PPy materials. Further, the FT-IR spectra of Py, PPy, and CNC@PPy are shown in Figure 1d. As observed, the adsorption bands at 3437 cm^–1^ can be attributed to the N-H stretching vibrations of the Py monomer [32]. The broad and moving absorption band peaking at 3365 cm^–1^ and 3243 cm^–1^ can be attributed to the polymerization of PPy [33,34]. And in the spectra of CNC@PPy, there was a typical adsorption peak at 3423 cm^–1^ that could be assigned to the stretching vibration of -OH group in CNC [35]. Moreover, the adsorption bands at 3191 cm^–1^ and 1264 cm^–1^ corresponded to the stretching vibrations of the N-H and -O- bonds in the CNC@PPy adsorbent, respectively [36]. From the above results of FT-IR measurement, it can be confirmed that the PPy coating with abundant groups was successfully polymerized on the surface of CNC, which could exhibit a reliable adsorption property toward Hg(II). In addition, Figure 1e shows the N_2_ adsorption–desorption isotherms of CNC@PPy, and its specific surface area was calculated to be 74.3 m^2^ g^−1^. Notably, a relatively high specific surface area can enhance the adsorption ability for Hg(II). 

**Figure 2:**
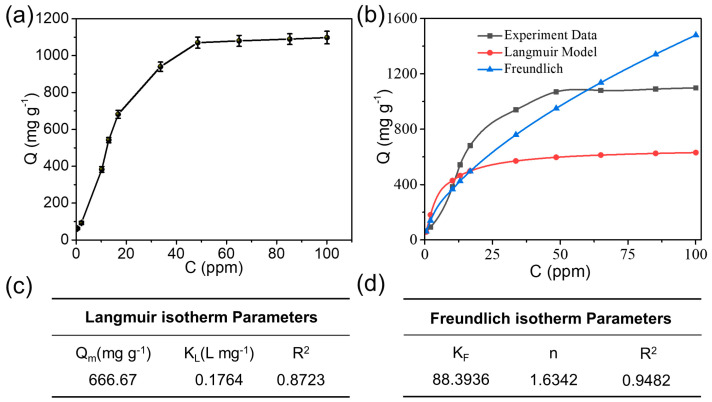
(**a**) Adsorption isotherms for the adsorption of Hg(II) on CNC@PPY, and (**b**) corresponding model of the adsorption isotherm. (**c**,**d**) Fitted parameters of two adsorption isotherms.

**Figure 3:**
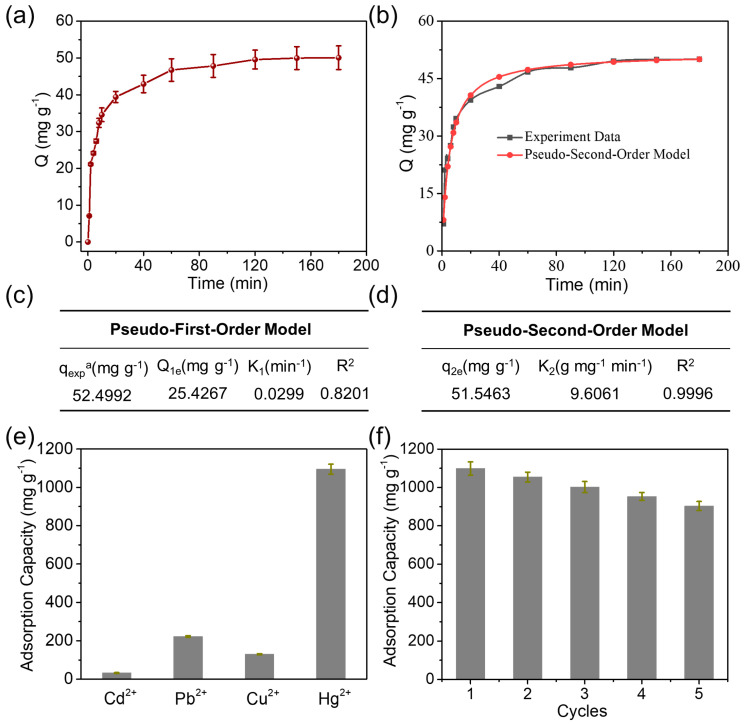
(**a**) Kinetic data and (**b**) the corresponding model of adsorption kinetics for the adsorption of Hg(II) on CNC@PPy. (**c**,**d**) Related parameters of two adsorption kinetics model. (**e**) Effect of co-anions on the Hg(II) adsorption. (**f**) Regeneration of CNC@PPy over 5 cycles.

The adsorption performance of Hg(II) on CNC@PPy was investigated first. Figure 2a shows the adsorption isotherms of CNC@PPy for Hg(II) at room temperature (25 °C) and a pH of 5.0. It clearly reveals that with the increase in Hg(II) concentration displayed a quickly enhanced equilibrium adsorption capacity toward Hg(II) in the low concentration region, and gradually saturated in the high concentration region (≥50 ppm), with a high adsorption capacity of 1095 mg g^–1^. The Langmuir model and Freundlich model were used to fit the adsorption properties of Hg(II), and the relevant experimental parameters are shown in Figure 2b–d. According to the fitted experimental data, the adsorption of Hg(II) by this material was consistent with the Freundlich adsorption isotherm model with R^2^ of 0.9482. This indicates that the adsorption of Hg(II) by the CNC/PPy adsorbent was a poly-molecular layer adsorption process and a surface adsorption behavior. The adsorption kinetic measurements were carried out at a solution presence of 10 ppm Hg(II) at pH = 5.0, and the adsorption amounts were determined at predetermined time intervals (Figure 3a). It was found that the adsorption reached the equilibrium status within 60 min for CNC@PPy. According to the fitting of adsorption kinetics model in Figure 3b, all adsorbent materials met the pseudo-second-order model [14,17,18,19]. The adsorption speed was the fastest, within 40 min, and the adsorption equilibrium could be reached. According to Figure 3b, the adsorption capacity increased with the increase of time. After 120 min of reaction, the adsorption of Hg(II) by the adsorbent material basically reached the equilibrium state. Moreover, according to the quasi-first-order and quasi-second-order fitting parameters in Figure 3c,d, the linear correlation coefficient obtained after fitting the quasi-second-order equation was 0.9996. The whole adsorption process of the CNC@PPy adsorbent for Hg(II) satisfied the quasi-second-order model, which indicates that the process is chemical adsorption, and the fitting parameters are shown in Figure 3c,d. These results prove the excellent adsorption properties of CNC@PPy, further implying the efficacy of PPy functionalization. The unique structure of CNC@PPy determines its superior adsorption properties for Hg(II). 

According to the fitting of adsorption kinetics model in Figure 3a–d, all adsorbent materials met the pseudo-second-order model. The adsorption speed was the fastest within 40 min, the adsorption equilibrium could be reached, and according to the figure, the adsorption capacity increased with the increase of time. After 120 min of reaction, the adsorption of Hg(II) by the adsorbent material basically reached the equilibrium state. Moreover, according to the quasi-first-order and quasi-second-order fitting parameters in Figure 3b,c, the linear correlation coefficient obtained after fitting the quasi-second-order equation was 0.9996. The whole adsorption process of the CNC/PPy adsorbent for Hg(II) satisfied the quasi-second-order model, which indicates that the process is chemical adsorption, and the fitting parameters are shown in Figure 3b,c below. The Langmuir model and Freundlich model were used to fit the adsorption properties of Hg(II), and the relevant experimental parameters were shown in Figure 3d–f. According to the fitted experimental data, the adsorption of Hg(II) by this product was consistent with the Freundlich adsorption isotherm model. This indicates that the adsorption of Hg(II) by CNC/PPy adsorbent is a single molecular layer adsorption process and a surface adsorption process [17,18,19]. The pseudo-second-order model with R^2^ = 0.948 (Figure 3d) better represented the adsorption process. This indicates that the rate controlling step of the adsorption is a chemical interaction process involving theCNC@ppy’s chelating function [19]. The parameters for the adsorption kinetic models are listed in Figure 3a–d.

To characterize the selectivity adsorption of CNC@PPy, a series of experiments were applied in a competitive system containing Hg(II) and other cations, including Cd(II), Pb(II), and Cu(II). The effects of introduction cations are exhibited in Figure 3e, exhibiting saturated adsorption capacities of 32.4 mg g^–1^, 223.6 mg g^–1^, and 129.5 mg g^–1^ for Cd(II), Pb(II), and Cu(II), respectively. The results show the Hg(II) adsorption capacity without obvious reduction with the presence of competitive cations. The result demonstrated that CNC@PPy possesses a high selectivity for Hg(II) in a complex competitive system. The property of cyclic adsorption behavior for a certain adsorbent is important for its underlying adaptability in wastewater remediation [14,19]. The reusability of the CNC@PPy for the adsorption of Hg(II) was investigated. For every adsorption measurement, 1.0 M HCl was applied to regenerate CNC@PPy by removing the adsorbed Hg(II) and rinsing with excess deionized water several times for the next cycle. As shown in Figure 3f, the adsorption ability of CNC@PPy towards Hg(II) could be maintained at 82.3% of the first adsorption capacity, likely due to a reduction in binding active sites following regeneration. Several characterizations were analyzed in detail to investigate the superior Hg(II) adsorption capability of CNC@PPy.

**Figure 4:**
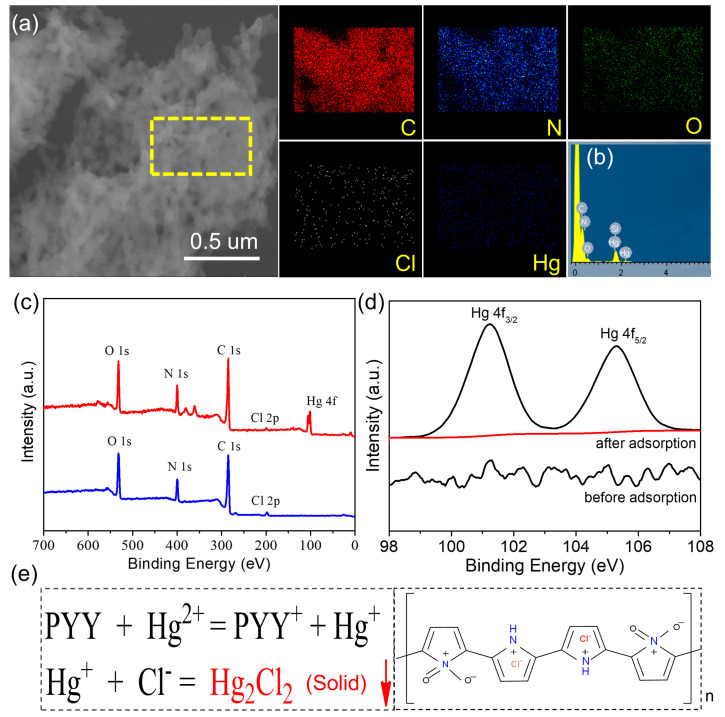
(**a**) SEM-EDS elemental mapping and (**b**) corresponding spectrum after adsorption of Hg^2+^ for CNC@PPy. (**c**) XPS survey spectra of CNC@PPy before and after adsorption of Hg(II). (**d**) High resolution XPS spectrum of Hg 4f after adsorption of Hg(II) for CNC@PPy. (**e**) Proposed adsorption mechanism of Hg(II) on CNC@PPy.

As shown in Figure 4a, after the CNC@PPy adsorption of Hg(II), its SEM-EDS elemental mapping and spectrum manifested the sample presence of C, N, O, Cl, and Hg elements. In addition, the corresponding EDS spectrum is shown in Figure 4b, confirming Hg(II) adsorption on the surface of CNC@PPy. Further, from the XPS survey spectra of CNC@PPy before and after the adsorption of Hg(II) (Figure 4c), the two samples both possessed C, N, O, and Cl elementals. In addition, the Hg-related signal appeared obviously on the sample of the CNC@PPy adsorption of Hg(II), suggesting that CNC@PPy can adsorb Hg(II). The high resolution XPS spectra of CNC@PPy before and after the adsorption of Hg(II) can confirm Hg(II) adsorption on the surface of CNC@PPy (Figure 4d). The XPS spectra of Hg 4f revealed two peaks at 101.3 eV and 105.3 eV, corresponding to Hg 4f_7/2_ and Hg 4f_5/2_, respectively. The Hg 4f_7/2_ obtained from the adsorbed Hg(II) ions (101.3 eV) was 0.2 eV higher than that of free Hg(II) (100.1 eV), confirming a strong interaction of the adsorbed Hg(II) with CNC@PPy. Such a strong binding energy shift suggests that the Hg(II) adsorption on CNC@PPy was due to strong chemical interactions rather than weak physical interactions. Moreover, the pyrrole monomer had polymerization via a simple hydrothermal with HCl as the protonation reagent to prepare Cl-doped PPy (Figure 4e). Thus, we speculate that the chemical interactions were obtained from the Hg_2_Cl_2_ precipitate between the Cl group in CNC@PPy and Hg(II) ions [13,14,18,34].

## 4. Conclusions

We have confirmed that PPy could be successfully coated on the surface of CNC using a facile and environmentally friendly polymerization approach, resulting in the CNC@PPy adsorbent displaying an excellent performance for the effective removal of Hg(II). The obtained CNC@PPy had a remarkably enhanced Hg(II) adsorption ability of 1095 mg g^−1^. The reason for the superior Hg(II) absorbability of CNC@PPy could be attributed to the introduction of the Cl functional group during the polymerization process under HCl conditions. In particular, the CNC@PPy showed an excellent re-usability, capable of retaining 82.3% of its original Hg(II) adsorption capacity after five consecutive adsorption cycles. The analysis of the experimental data showed that the Freundlich model outperformed the Langmuir model in describing the isotherms. Similarly, the pseudo-second order kinetic model exhibited a stronger correlation with the experimental data compared to the pseudo-first order model. The findings of this work open a path to upgrade this agricultural by-product into value products for environmental remediation applications.

## Data Availability

Not applicable.

## Supplementary Materials

The following supporting information can be downloaded at: https://www.mdpi.com/article/10.3390/polym15122735/s1, Supporting Information.

## References

[1] Tanpichai S., Boonmahitthisud A., Soykeabkaew N., Ongthip L. (2022). Review of the Recent Developments in all-Cellulose NanoComposites: Properties and Applications. Carbohydr. Polym..

[2] Rol F., Belgacem M.N., Gandini A., Bras J. (2019). Recent Advances in Surface-Modified Cellulose Nanofibrils. Prog. Polym. Sci..

[3] Yang G., Kong H., Chen Y., Liu B., Zhu D., Gao L., Wei G. (2022). Recent Advances in the Hybridization of Cellulose and Carbon Nanomaterials: Interactions, Structural Design, Functional Tailoring, and Applications. Carbohydr. Polym..

[4] Hokkanen S., Bhatnagar A., Sillanpaa M. (2016). A Review on Modification Methods to Cellulose-based Adsorbents to Improve Adsorption Capacity. Water Res..

[5] Fakhre N.A., Ibrahim B.M. (2018). The use of New Chemically Modified Cellulose for Heavy Metal ion Adsorption. J. Hazard. Mater..

[6] Wohlhauser S., Delepierre G., Labet M., Morandi G., Thielemans W., Weder C., Zoppe J.O. (2018). Grafting Polymers from Cellulose Nanocrystals: Synthesis, Properties, and Applications. Macromolecules.

[7] Sun B., Yuan Y., Li H., Li X., Zhang C., Guo F., Liu X., Wang K., Zhao X.S. (2019). Waste-cellulose-derived porous Carbon Adsorbents for Methyl Orange Removal. Chem. Eng. J..

[8] Aoudi B., Boluk Y., Gamal El-Din M. (2022). Recent Advances and Future Perspective on Nanocellulose-based Materials in Diverse Water Treatment Applications. Sci. Total Environ..

[9] Kargarzadeh H., Mariano M., Gopakumar D., Ahmad I., Thomas S., Dufresne A., Huang J., Lin N. (2018). Advances in Cellulose Nanomaterials. Cellulose.

[10] Ding Q., Li C., Wang H., Xu C., Kuang H. (2021). Electrochemical Detection of Heavy Metal Ions in Water. Chem. Commun..

[11] Ge Y., Li Z. (2018). Application of Lignin and its Derivatives in Adsorption of Heavy Metal Ions in Water: A Review. ACS. Sustain. Chem. Eng..

[12] Sarma G.K., Sen Gupta S., Bhattacharyya K.G. (2019). Nanomaterials as Versatile Adsorbents for Heavy Metal Ions in Water: A review. Environ. Sci. Pollut. Res..

[13] Geng Z., Zhang H., Xiong Q., Zhang Y., Zhao H., Wang G. (2015). A fluorescent Chitosan Hydrogel Detection Platform for the Sensitive and Selective Determination of Trace Mercury(II) in Water. J. Mater. Chem. A.

[14] Xu G., Wang L., Xie Y., Tao M., Zhang W. (2018). Highly Selective and Efficient Adsorption of Hg^2+^ by a Recyclable Aminophosphonic acid Functionalized Polyacrylonitrile Fiber. J. Hazard. Mater..

[15] Bethke K., Palantöken S., Andrei V., Roß M., Raghuwanshi V.S., Kettemann F., Greis K., Ingber T.T.K., Stückrath J.B., Valiyaveettil S. (2018). Functionalized Cellulose for Water Purification, Antimicrobial Applications, and Sensors. Adv. Funct. Mater..

[16] Carpenter A.W., de Lannoy C.F., Wiesner M.R. (2015). Cellulose Nanomaterials in Water Treatment Technologies. Environ. Sci. Technol..

[17] Arsenie T., Cara I.G., Popescu M.C., Motresu I., Bulgariu L. (2022). Evaluation of the Adsorptive Performances of Rapeseed Waste in the Removal of Toxic Metal Ions in Aqueous Media. Water.

[18] Xu G., Zhao Y., Hou L., Cao J., Tao M., Zhang W. (2017). A Recyclable Phosphinic Acid Functionalized Polyacrylonitrile Fiber for Selective and Efficient Removal of Hg^2+^. Chem. Eng. J..

[19] Zhou R., Xu W., Liu P., Zhao S., Xu G., Xiong Q., Zhang W., Zhang C., Ye X. (2023). Synthesis of FeOOH-Loaded Aminated Polyacrylonitrile Fiber for Simultaneous Removal of Phenylphosphonic Acid and Phosphate from Aqueous Solution. Polymers.

[20] Mohammed N., Grishkewich N., Tam K.C. (2018). Cellulose Nanomaterials: Promising Sustainable Nanomaterials for AppliCation in Water/wastewater Treatment Processes. Environ. Sci. Nano.

[21] Bulgariu D., Nemeş L., Ahmad I., Bulgariu L. (2023). Isotherm and Kinetic Study of Metal Ions Sorption on Mustard Waste Biomass Functionalized with Polymeric Thiocarbamate. Polymers.

[22] Chen Y., Zhang J., Cohen Y. (2022). Fouling Resistant and Performance Tunable Ultrafiltration Membranes via Surface Graft Polymerization Induced by Atmospheric Pressure Air Plasma. Sep. Purif. Technol..

[23] Hwang I.-T., Han D.-S., Sohn J.-Y., Shin J., Choi J.-H., Jung C.-H. (2022). Preparation and Cesium Adsorption Behavior of Prussian blue-based Polypropylene Nonwoven Fabric by Surfactant-assisted Aqueous Preirradiation Graft Polymerization. Radiat. Phys. Chem..

[24] Zhang J., Chen H., Chen Z., He J., Shi W., Liu D., Chi H., Cui F., Wang W. (2016). Microstructured Macroporous Adsorbent Composed of Polypyrrole Modified Natural Corncob-core Sponge for Cr(vi) Removal. RSC Adv..

[25] Zhang L., Niu W., Sun J., Zhou Q. (2020). Efficient removal of Cr (VI) from water by the uniform fiber ball loaded with polypyrrole: Static adsorption, dynamic adsorption and mechanism studies. Chemosphere.

[26] Rong Y., Yan W., Wang Z., Hao X., Guan G. (2022). An Electroactive Montmorillonite/polypyrrole Ion Exchange film: Ultrahigh Uptake Capacity and Ion Selectivity for Rapid Removal of Lead Ions. J. Hazard. Mater..

[27] Du L., Gao P., Meng Y., Liu Y., Le S., Yu C. (2020). Highly Efficient Removal of Cr(VI) from Aqueous Solutions by Polypyrrole/Monodisperse Latex Spheres. ACS Omega.

[28] Das R., Giri S., Muliwa A.M., Maity A. (2017). High-Performance Hg(II) Removal Using Thiol-Functionalized Polypyrrole (PPy/MAA) Composite and Effective Catalytic Activity of Hg(II)-Adsorbed Waste Material. ACS Sustain. Chem. Eng..

[29] Xiao S., Xiong Q., Zhou H., Zhang Y., Wang G., Zhang H., Zhao H. (2016). Oxoacetohydrazide-Functionalized Cellulose with Enhanced Adsorption Performance. J. Appl. Polym. Sci..

[30] Rehman N., de Miranda M.I.G., Rosa S.M.L., Pimentel D.M., Nachtigall S.M.B., Bica C.I.D. (2014). Cellulose and Nanocellulose from Maize Straw: An Insight on the Crystal Properties. J. Polym. Environ..

[31] Moharrami P., Motamedi E. (2020). Application of Cellulose Nanocrystals Prepared from Agricultural Wastes for Synthesis of Starch-based Hydrogel Nanocomposites: Efficient and Selective Nanoadsorbent for Removal of Cationic Dyes from Water. Bioresour. Technol..

[32] Saleh T.A., Tuzen M., Sarı A., Altunay N. (2022). Factorial Design, Physical Studies and Rapid Arsenic Adsorption Using Newly Prepared Polymer Modified Perlite adsorbent. Chem. Eng. Res. Des..

[33] González-Casamachin D.A., De la Rosa J.R., Lucio-Ortiz C.J., De Rio D.A.D.H., Martínez-Vargas D.X., Flores-Escamilla G.A., Guzman N.E.D., Ovando-Medina V.M., Moctezuma-Velazquez E. (2019). Visible-light Photocatalytic Degradation of Acid Violet 7 Dye in a Continuous Annular Reactor Using ZnO/PPy Photocatalyst: Synthesis, Characterization, Mass Transfer Effect Evaluation and Kinetic Analysis. Chem. Eng. J..

[34] Farid S., Qiu W., Zhao J., Song X., Mao Q., Ren S., Hao C. (2020). Improved OER Performance of Co_3_O_4_/N-CNTs Derived from Newly Designed ZIF-67/PPy NTs Composite. J. Electroanal. Chem..

[35] Zhou B., Li J., Liu W., Jiang H., Li S., Tan L., Dong L., She L., Wei Z. (2020). Functional-Group Modification of Kraft Lignin for Enhanced Supercapacitors. ChemSusChem.

[36] Wang R.-P., Yang B., Fu Q., Zhang Y., Zhu R., Dong X.-R., Zhang Y., Wang B., Yang J.-L., Luo Y. (2021). Raman Detection of Bond Breaking and Making of a Chemisorbed Up-Standing Single Molecule at Single-Bond Level. J. Phys. Chem. Lett..

