# Supplementary material for: Polypyrrole-Modified Nanocellulose Exhibits Superior Performance for Hg(II) Adsorption
Source: Polymers (Basel). 2023 Jun 19;15(12):2735. doi: 10.3390/polym15122735 (PMC10302129; doi:10.3390/polym15122735)
Supplement: Supplementary file 1 [file polymers-15-02735-s001.zip › polymers-2425519-supplementary.pdf]

Supporting Information for

Polypyrrole-Modified Nanocellulose Exhibits Superior Performance for Hg(II) Adsorption

Qizhong Xiong [1,2,*], Lei Zhang [1,2], Zijun Zhu [1,2], Gang Xu [1,2], Jianyuan Jing [1,2], Weifeng Zhang [1], Chaochun Zhang [1] and Xinxin Ye [1,2,*]

[1] Anhui Province Key Lab of Farmland Ecological Conservation and Pollution Prevention, Anhui Province Engineering and Technology Research Center of Intelligent Manufacture and Efficient Utilization of Green Phosphorus Fertilizer, College of Resources and Environment, Anhui Agricultural University, Hefei 230036, China

[2] Key Laboratory of JiangHuai Arable Land Resources Protection and Eco-Restoration, Ministry of Natural Resources, College of Resources and Environment, Anhui Agricultural University, Hefei 230036, China

* Correspondence: qzxiong@ahau.edu.cn (Q.X.); xxye@ahau.edu.cn (X.Y.)

For the adsorption isotherm and kinetic study, the following equations were used.

Isotherm equations:

Langmuir: $\frac{1}{q_e}=\frac{1}{q_m}+\frac{1}{q_m K_1 C_e}$;

Freundlich: $\ln q_e = \ln K_f + \frac{1}{n}\ln C_e$;

Temkin: $q_e = B\ln A_T + B\ln C_e$, Where $B=\frac{RT}{b_T}$;

Dubinin–Radushkevich isotherm model: $q_e=(q_s)exp(-K_{ad}\mathcal{E}^2)$, where $\mathcal{E}$= RT $\ln(1+\frac{1}{C_e})$, $E=\frac{1}{\sqrt{2K_{ad}}}$;

Kinetic equations:

pseudo-first order: $\log(q_e-q_t)=\log q_e-\frac{k_1}{2.303}t$;

pseudo-second order: $\frac{t}{q_t}=\frac{1}{k_2 q_e^2}+\frac{1}{q_e}t$;

Intraparticle diffusion model: $q_t=k_p t^{1/2}$;

where q_e is the equilibrium sorption capacity (mg/g), q_m is the maximum adsorption capacity of the sorbent (mg/g), C_e is the equilibrium concentration (mg/L), K_l is the Langmuir adsorption constant (L/mg), K_f is the Freundlich isotherm constant (mg/g), n is the adsorption intensity, A_T is the Temkin isotherm equilibrium binding constant (L/g), b_T is the Temkin isotherm constant (J/mol), B is the constant related to the heat of sorption, T is the temperature at 298 (K), R is the universal gas constant (8.314 J $mol^{-1}K^{-1}$), q_s is Dubinin–Radushkevich model constant (mg/g), K_{ad} is Dubinin–Radushkevich isotherm constant (mol^2/kJ^2), ε is the Polanyi potential, E is mean free energy, t is time (min), k_1 (min^{-1}) and k_2 (mg g^{-1} min^{-1}) are the first and second-order rate constants, respectively, k_p is the kinetics rate constant of intraparticle diffusion model ($g\cdot mg^{-1}\cdot min^{-0.5}$), and q_t is the amount of heavy metal ion (mg/g) adsorbed at time

The related calculating formula and fitting model: The removal percentage (%) was calculated by the following formula:

Removal percentage = $\frac{c_0-c_e}{c_0}\times 100\%$ (1)

where c_0 and c_e (mg·L^{-1}) represent the initial and equilibrium concentrations of Cr(VI) ions.

The capacity of absorbent on chromium(VI) removal q_e (mg·g^{-1}) was calculated by the following formula:

Removal capacity: $q_e = \frac{(c_0-c_e)m}{V}$ (2)

where c_0 and c_e (mg·L^{-1}) represent the initial and equilibrium concentrations of Cr(VI) ions, V (L) is the volume of Cr(VI) solution and m (g) is the quality of adsorbent inserted into the target solution.

In order to evaluate adsorption isotherm, both the Langmuir model and Freundlich model were employed to fit adsorption isotherm data. Their linear equations are as follows:

Langmuir model: $\frac{c_e}{q_e} = \frac{c_e}{q_{max}} + \frac{1}{K_L q_{max}}$ (3)

Freundlich model: $\log q_e = \log K_F + \frac{\log c_e}{n}$ (4)

where q_e (mg·g^{-1}) is the amount of chromium adsorbed at equilibrium, q_{max} (mg·g^{-1}) is the Langmuir maximum adsorption capacity, c_e (mg·L^{-1}) denotes the equilibrium concentration of the solution, K_L (L·mg^{-1}) and K_F (mg·g^{-1}) represent the Langmuir and Freundlich constants respectively, and n is an empirical parameter related to the intensity of adsorption.

To evaluate adsorption kinetics process of 1T-MoS_2, the kinetic curves were fitted with the pseudo-first-order kinetic model and pseudo-second-order kinetic model, which can be described by the following equations, respectively:

Pseudo-first-order kinetic model: $\log(q_e - q_t) = \log q_e - k_1$ (5)

Pseudo-second-order kinetic model: $\frac{t}{q_t} = \frac{1}{k_2 q_e^2} + \frac{t}{q_e}$ (6)

where q_e and q_t (mg·g^{-1}) are the amounts of chromium adsorbed at the equilibrium and

at time t (min), respectively. k_1 (min^{-1}) and k_2 ($g \cdot mg^{-1} \cdot min^{-1}$) are the kinetic rate constants for the pseudo-first-order and pseudo-second-order kinetic models, respectively.
